# Cardiometabolic risk factors among children who are affected by overweight, obesity and severe obesity

**DOI:** 10.3389/fpubh.2023.1097675

**Published:** 2023-04-27

**Authors:** Ahmad Kamil Nur Zati Iwani, Muhammad Yazid Jalaludin, Farah Aqilah Roslan, Fazliana Mansor, Fuziah Md Zain, Janet Yeow Hua Hong, Ruziana Mona Wan Mohd Zin, Abqariyah Yahya, Zahari Ishak, Rusidah Selamat, Abdul Halim Mokhtar

**Affiliations:** ^1^Endocrine and Metabolic Unit, Institute for Medical Research, Ministry of Health Malaysia, Setia Alam, Malaysia; ^2^Department of Paediatrics, Faculty of Medicine, University Malaya, Kuala Lumpur, Malaysia; ^3^Department of Paediatrics, Hospital Putrajaya, Ministry of Health Malaysia, Putrajaya, Malaysia; ^4^Department of Social and Preventive Medicine, Faculty of Medicine, University Malaya, Kuala Lumpur, Malaysia; ^5^Department of Educational Psychology and Counselling, Faculty of Education, University Malaya, Kuala Lumpur, Malaysia; ^6^Nutrition Division, Ministry of Health Malaysia, Putrajaya, Malaysia; ^7^Department of Sports Medicine, Faculty of Medicine, University Malaya, Kuala Lumpur, Malaysia

**Keywords:** childhood obesity, severely obese, cardiometabolic risks, metabolic syndrome, acanthosis nigricans

## Abstract

**Background:**

The increasing severity of obesity is expected to lead to more serious health effects. However, there is limited information on the prevalence and clinical characteristics of cardiometabolic risk factors in severely children affected by obesity in Malaysia. This baseline study aimed to investigate the prevalence of these factors and their association with obesity status among young children.

**Methods:**

In this study, a cross-sectional design was employed using the baseline data obtained from the My Body Is Fit and Fabulous at school (MyBFF@school) intervention program involving obese school children. Obesity status was defined using the body mass index (BMI) *z*-score from the World Health Organization (WHO) growth chart. Cardiometabolic risk factors presented in this study included fasting plasma glucose (FPG), triglycerides (TGs), total cholesterol, high-density lipoprotein cholesterol (HDL-C), low-density lipoprotein cholesterol (LDL-C), blood pressure, acanthosis nigricans, insulin resistance (IR), and MetS. MetS was defined using the International Diabetes Federation (IDF) 2007 criteria. Descriptive data were presented accordingly. The association between cardiometabolic risk factors, such as obesity status, and acanthosis nigricans with MetS was measured using multivariate logistic regression, which was adjusted for gender, ethnicity, and strata.

**Results:**

Out of 924 children, 38.4% (*n* = 355) were overweight, 43.6% (*n* = 403) were obese, and 18% (*n* = 166) were severely obese. The overall mean age was 9.9 ± 0.8 years. The prevalence of hypertension, high FPG, hypertriglyceridemia, low HDL-C, and the presence of acanthosis nigricans among severely children affected by obesity was 1.8%, 5.4%, 10.2%, 42.8%, and 83.7%, respectively. The prevalence of children affected by obesity who were at risk of MetS in <10-year-old and MetS >10-year-old was observed to be similar at 4.8%. Severely children affected by obesity had higher odds of high FPG [odds ratio (OR) = 3.27; 95% confdence interval (CI) 1.12, 9.55], hypertriglyceridemia (OR = 3.50; 95%CI 1.61, 7.64), low HDL-C (OR = 2.65; 95%CI 1.77, 3.98), acanthosis nigricans (OR = 13.49; 95%CI 8.26, 22.04), IR (OR = 14.35; 95%CI 8.84, 23.30), and MetS (OR = 14.03; 95%CI 3.97, 49.54) compared to overweight and children affected by obesity. The BMI z-score, waist circumference (WC), and percentage body fat showed a significant correlation with triglycerides, HDL-C, the TG: HDL-C ratio, and the homeostatic model assessment for IR (HOMA-IR) index.

**Conclusions:**

Severely children affected by obesity exhibit a higher prevalence of and are more likely to develop cardiometabolic risk factors compared to overweight and children affected by obesity. This group of children should be monitored closely and screened periodically for obesity-related health problems to institute early and comprehensive intervention.

## 1. Introduction

There is a high prevalence of obesity worldwide, affecting both adults and children (1, 2). The most recent national prevalence of overweight and obesity among Malaysian children aged 5–17 years was 15.0% and 14.8%, respectively (3). Despite an increase in the prevalence of obesity over the past 15 years, data on the impact of overweight, obesity, and severe obesity in young children in the world, including Malaysia, are still scarce. In some countries, the increase in childhood obesity preceded the increase in adult obesity (4). This high prevalence of obesity has led to heightened awareness and concerns relevant to many significant health problems, such as type 2 diabetes mellitus (T2DM), liver disease, hyperlipidemia, and cardiovascular disease (CVD), which consequently increase healthcare costs (5, 6). Several studies have shown that severe obesity was associated with a greater risk of weight-related complications, including abnormal lipid levels, blood glucose levels, and increased blood pressure (7, 8).

A systematic review and meta-analysis of 63 studies examining 49,200 children found that obesity was associated with significantly worse risk parameters for CVD in school-aged children (9). However, the finding was obtained from studies conducted in a highly developed country. Studies on cardiometabolic risk factors and their association with obesity status in school-aged children are still insufficiently explored in low- and middle-income countries, such as Malaysia. Cardiometabolic risk factors associated with obesity may include acanthosis nigricans, insulin resistance (IR), elevated blood pressure, and dyslipidemia, which may later lead to CVD and T2DM. A recent study by Garcia et al. (10) found that children affected by obesity with grade 3 acanthosis nigricans (the degree of severity in the neck by Burke's scale) were associated with increased waist circumference (WC), triglycerides (TGs), low-density lipoprotein cholesterol (LDL-C), total cholesterol, and homeostatic model assessment for IR (HOMA-IR), which might lead to vascular alterations. Similarly, our group also previously found that the majority of children with acanthosis nigricans (43%) were in the upper tertiles of the ratio of triglycerides to high-density lipoprotein cholesterol (TG: HDL-C) (11).

In addition to acanthosis nigricans, the metabolic syndrome (MetS), which encloses the clustering of cardiometabolic disorders, such as abdominal obesity, elevated fasting plasma glucose (FPG), dyslipidemia, and hypertension, is also an important tool for identifying individuals who are at a high risk of T2DM and CVD (12, 13). A previous study showed that the prevalence of obesity among Malaysian children diagnosed with MetS was 10% (14). The individual risk factors of MetS (abdominal obesity, high triglycerides, low HDL-C, high blood pressure, and high FPG) were found to be higher in children affected by obesity than in children with normal weight (14). The findings of this current study may provide a comparison of obesity status among school-aged children in Malaysia to previous studies and help develop effective policy measures to prevent obesity. Therefore, this study aimed to determine the prevalence of cardiometabolic risk factors and their association with obesity status among children who participated in My Body Is Fit and Fabulous at school (MyBFF@school) programs.

## 2. Methods

### 2.1. Study design and sampling

This study was a cross-sectional study, which used the baseline data of overweight, obesity, and severe obesity among young children who participated in the MyBFF@school phase II program. This programs was a school-based lifestyle intervention program that included nutritional–physical activity and psychology modules specifically designed for children affected by obesity (15). Between January and early March 2016, children and adolescents from 23 Malaysian Public Schools were screened. Eligible children were invited to participate in the intervention program. A written informed consent was obtained from parents or guardians, and the assent form was signed by the participating child.

### 2.2. Study participants

All children affected by obesity classified as overweight, obese, and severely obese according to the World Health Organization (WHO) body mass index (BMI) chart (16) using the BMI z-score criteria were eligible for this study. Children affected by obesity with a physical or mental impairment, with medical conditions that prevent him/her from participating in moderate-to-vigorous physical activity, with comorbidities that may interfere with the study (such as diagnosed T2DM, hypertension, nephritic syndrome, epilepsy, congenital heart disease, and skeletal anomalies), and on steroids, anti-epileptics, and methylphenidate were excluded from this study.

**Figure 1:**
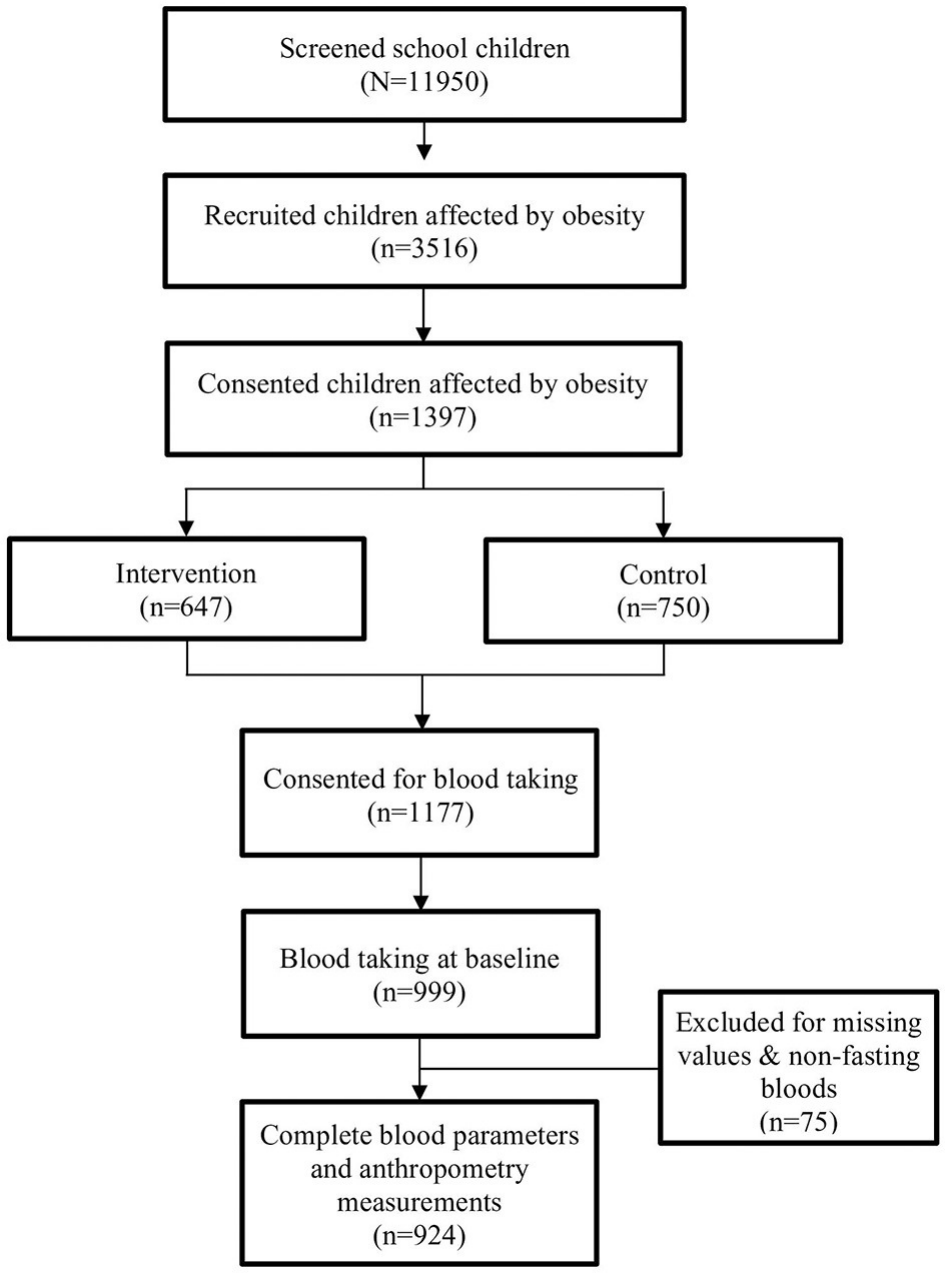
Flow chart for My Body is Fit and Fabulous at School (MyBFF@school) study at baseline.

A total of 11,950 primary school children aged 8–11 years were screened, and 3,516 (29.4%) children were eligible to participate. Of the 3,516 children, 1,397 (39.7%) gave their consent to participate in this study, with 647 of them in the intervention group and the remaining 750 children in the control group. At baseline, children in both groups underwent a similar screening that was performed in the morning because children were assessed during fasting. In this paper, 924 children with complete anthropometric measurements and fasting blood parameters at baseline were analyzed (Figure 1).

### 2.3. Anthropometric measurements

These measurements were taken two times, and their average was used in the data analysis. BMI was calculated by dividing weight in kilograms by the square of height in meters (kg/m^2^). The weight category was grouped based on the WHO BMI chart (16) using the BMI z-score criteria, which is overweight, obese, and severely obese. The BMI *z*-score was calculated using the WHO AnthroPlus 2007 software, with an indication of overweight > +1.0 standard deviation (SD), obese > +2.0 SD, and severely obese > +3.0 SD (an extended cutoff from the WHO growth curve) (17, 18). WC was measured using a non-extensible tape (Seca 201, Germany) to the nearest 0.1 cm at the midpoint between the lowest rib and the iliac crest. Body weight and the percentage of body fat (PBF) were measured using a bioelectrical impedance analysis (InBody 720, BioSpace, Korea). Children were required to stand barefoot on the body composition analyzer while holding electrode hand holders for 3 min.

### 2.4. Clinical measurements

Blood pressure reading was gathered manually by trained staff using a mercury sphygmomanometer (Accoson, UK) with an appropriate cuff size for each individual. Children were in a sitting position with their right upper arm positioned at the heart level and their feet flat on the ground. The reading was taken two times at 5-min intervals to improve accuracy, and the mean was recorded. Physical examination for acanthosis nigricans at the nape of the neck was performed by trained medical officers/pediatricians.

A venous blood sample (after a minimum of 8 h fasting) was collected by trained nurses/medical officers. To measure blood glucose, 2 ml of blood was collected in a sodium fluoride tube, and to measure the lipid profile (total cholesterol, triglycerides, HDL-C, and LDL-C), another 5 ml in a plain tube test tube was collected. All blood samples were labeled and transported in an ice box to the central laboratory of the Institute for Medical Research within 2 h of collection. Serum total cholesterol, triglycerides, HDL-C, and LDL-C were assessed by the enzymatic colorimetric method, while the FPG level was determined by the enzymatic oxidation colorimetric method. All samples were analyzed by Randox Laboratories (Antrim, UK) using an autochemical analyzer, Dirui CS-400.

Children with MetS were determined according to the International Diabetes Federation (IDF) 2007 definition, which was defined as having a WC of more than the 90th percentile with the presence of at least 2 other clinical characteristics (triglycerides ≥ 1.7 mmol/L, HDL-C < 1.03 mmol/L, blood pressure ≥ 130/85 mmHg, and FPG ≥ 5.6 mmol/L) (19). These criteria are set for children over 10 years of age. Therefore, children who were <10 years of age and fulfilled the above criteria were classified as being at risk of MetS.

The IR status was based on the HOMA-IR calculated by multiplying the value of fasting plasma insulin and FPG and then dividing the multiplied value by 22.5 (20). In prepubertal children, a score of HOMA-IR of ≥2.6 (21) was classified as IR and of <2.6 as insulin sensitive. In pubertal children, a score of HOMA-IR of ≥ 4.0 was categorized as IR, while a score of <4.0 was categorized as insulin sensitive (22).

### 2.5. Statistical analysis

All data analyses were performed using the IBM Statistical Package for the Social Sciences (SPSS) version 22. The normality test of continuous data was determined using the Kolmogorov–Smirnov test. Continuous data were presented as mean and SD, while categorical data were presented as frequency and percentage. Comparison of the means was determined using the Kruskal–Wallis test for non-normally distributed variables. The chi-squared test was used to compare differences between the categorical data. The association between cardiometabolic risk factors by obesity status and acanthosis nigricans with MetS was measured using multivariate logistic regression and adjusted for gender, ethnicity, and strata. Spearman's correlation test was used to correlate anthropometry (BMI z-score, WC, and PBF) with MetS components and IR. A *p*-value of <0.05 was considered statistically significant for all analyses.

## 3. Results

Baseline sociodemographics by obesity status are presented in Table 1. The final number of children with complete data was 924, ranging in age from 8.0 to 11 years, with a mean (SD) age of 9.8 (0.84) and a median age of 9.9 years. In total, 52.5% were men and 47.5% were women. The majority of children were Malay (75.4%), followed by Chinese (12.4%), Indians (10.4%), and other ethnic groups (1.7%). Of the 924 children, 38.4% were overweight, 43.6% were obese, and 18.0% of them were severely obese. No significant differences were observed between school locations.

**Table 1:** Sociodemographic at baseline by obesity status.

**Features**	**Overweight (*n* = 355)**	**Obese (*n* = 403)**	**Severely obese (*n* = 166)**	***P*** **-value**	**Total (*N* = 924)**
Total (%)	38.4	43.6	18.0	–	100.0
**Sociodemographic**					
**Age**					
Overall, mean ± SD	9.9 ± 0.9	9.9 ± 0.8	9.7 ± 0.9	0.009	9.9 ± 0.8
<10 years old, median (p25, p75)	9.0 (8.6, 9.6)	9.3 (8.8, 9.7)	8.9 (8.6, 9.4)	0.024	9.1 (8.7, 9.6)
≥10 years old, median (p25, p75)	10.6 (10.3, 10.9)	10.5 (10.2, 10.8)	10.5 (10.2, 10.7)	NS	10.5 (10.3, 10.8)
**Gender**, ***n*** **(%)**					
Boys	159 (44.8)	212 (52.6)	114 (68.7)	<0.001	485 (52.5)
Girls	196 (55.2)	191 (47.4)	52 (31.3)		439 (47.5)
**Pubertal status**, ***n*** **(%)**					
**Boys**					
Pre-pubertal	137 (85.6)	191 (90.5)	98 (86.0)	NS	426 (87.8)
Pubertal	23 (14.4)	20 (9.5)	16 (14.0)		59 (12.2)
**Girls**					
Pre-pubertal	137 (85.6)	191 (90.5)	98 (86.0)	NS	426 (87.8)
Pubertal	23 (14.4)	20 (9.5)	16 (14.0)		59 (12.2)
**Ethnicity**, ***n*** **(%)**					
Malay	263 (74.1)	301 (74.7)	133 (80.1)	NS	697 (75.4)
Chinese	46 (13.0)	53 (13.2)	16 (9.6)		115 (12.4)
Indian	41 (11.5)	42 (10.4)	13 (7.8)		96 (10.4)
Others	5 (1.4)	7 (1.7)	4 (2.4)		16 (1.7)
**School location**, ***n*** **(%)**					
Urban	186 (52.4)	242 (60)	96 (57.8)	NS	524 (56.7)
Rural	169 (47.6)	161 (40)	70 (42.2)		400 (43.3)

NS, not significant; BMI, body mass index; HDL-C, high-density lipoprotein cholesterol; LDL-C, low-density lipoprotein cholesterol.

Table 2 presents anthropometric, clinical, and biochemical profiles at baseline by obesity status. As data on continuous variables were not normally distributed, median results were reported. The median of both BMI and the BMI z-score increased as a function of obesity status. Similarly, median WC and PBF increased as a function of obesity status. There was no significant difference in the blood pressure measurement. From the biochemical profile, severely children affected by obesity demonstrated higher triglycerides, higher median (0.9 mmol/l) and lower HDL-C, and lower median levels (1.0 mmol/l) compared to overweight and children affected by obesity.

**Table 2:** Anthropometric measures, clinical measures, and biochemical profile at baseline by obesity status.

**Features**	**Overweight (*n* = 355)**	**Obese (*n* = 403)**	**Severely obese (*n* = 166)**	***P*** **-value**	**Total (*N* = 924)**
**Anthropometric measures**					
BMI (kg/m^2^), median (p25, p75)	20.4 (19.5, 21.3)	24.0 (22.9, 25.7)	29.1 (27.3, 31.3)	<0.001	23.2 (20.8, 25.9)
BMI z score median (p25, p75)	1.53 (1.29, 1.78)	2.44 (2.21, 2.74)	3.49 (3.24, 4.01)	<0.001	2.23 (1.69, 2.83)
Waist circumference (cm), median (p25, p75)	66.6 (63.4, 71.0)	77.0 (72.9, 81.2)	87.2 (81.4, 94.4)	<0.001	74.3 (68.0, 81.2)
Percentage of body fat (%), median (p25, p75)	32.6 (28.8, 35.7)	39.8 (36.7, 42.6)	45.7 (43.2, 48.3)	<0.001	37.8 (33.3, 42.8)
**Clinical measures**					
**Blood pressure**					
Systolic (mmHg), median (p25, p75)	100.0 (91.0, 105.0)	100.0 (92.0, 104.0)	100.9 (91.0, 105.0)	NS	100.0 (92.0, 104.0)
Diastolic (mmHg), median (p25, p75)	60.0 (57.0, 69.0)	61.0 (55.0, 69.0)	60.0 (57.8, 69.0)	NS	60.0 (57.0, 69.0)
**Biochemical profile**					
Fasting plasma glucose (mmol/L), median (p25, p75)	4.7 (4.5, 4.9)	4.7 (4.5, 4.9)	4.7 (4.5, 5.0)	NS	4.7 (4.5, 4.9)
Total cholesterol (mmol/L), median (p25, p75)	4.1 (3.7, 4.6)	4.1 (3.7, 4.6)	4.0 (3.6, 4.5)	NS	4.1 (3.7, 4.6)
Triglycerides (mmol/L), median (p25, p75)	0.7 (0.5, 1.0)	0.9 (0.7, 1.2)	0.9 (0.7, 1.2)	<0.001	0.8 (0.6, 1.1)
HDL-C (mmol/L), median (p25, p75)	1.1 (1.0, 1.3)	1.1 (0.9, 1.2)	1.0 (0.9, 1.2)	<0.001	1.1 (0.9, 1.2)
LDL-C (mmol/L), median (p25, p75)	2.8 (2.4, 3.3)	2.8 (2.4, 3.4)	2.9 (2.4,3.4)	NS	2.8 (2.4, 3.4)

NS, not significant; BMI, body mass index; HDL-C, high-density lipoprotein cholesterol; LDL-C, low-density lipoprotein cholesterol.

Table 3 presents the prevalence of cardiometabolic risk factors by obesity status. It was found that the prevalence of all cardiometabolic risks was significantly higher with a greater degree of obesity. Severely children affected by obesity had a significantly higher prevalence of hypertension, high FPG, high triglycerides, and low HDL-C with frequencies of 1.8%, 5.4%, 10.2%, and 42.8%, respectively, compared to overweight and children affected by obesity. The presence of acanthosis nigricans was significantly more common in severely children affected by obesity, which was 83.7% as compared to 58.8% in children affected by obesity and 31.3% in overweight children. Severely children affected by obesity also had the highest prevalence of IR using the HOMA-IR calculation, which was 72.8% and exhibited the highest value of the TG: HDL-C ratio. All severely children affected by obesity had WC above the 90th percentile (*p* < 0.001). Severely children affected by obesity had the highest PBF (45.5%), followed by 39.8% in children affected by obesity and 32.6% in overweight children. For children over 10 years of age, the prevalence of MetS increased significantly with the degree of obesity, which was 0.3%, 2.7%, and 4.8% in overweight, obese, and severely children affected by obesity, respectively. A similar prevalence of being at risk of MetS was also observed among children <10 years of age of 0.6%, 2.0%, and 4.8% in overweight, obese, and severely children affected by obesity, respectively.

**Table 3:** Prevalence of cardiometabolic risk factors by obesity status.

**Features**	**Overweight (*n* = 355), *n* (%)**	**Obese (*n* = 403), *n* (%)**	**Severely obese (*n* = 166), *n* (%)**	***P*** **-value**
Waist circumference (cm) ≥90^th^ percentile	126 (35.5)	343 (85.1)	166 (100.0)	<0.001
Percentage body fat, mean (sd)	32.2 (4.8)	39.8 (4.4)	45.5 (4.2)	<0.001^a^
Hypertension (≥130/85 mmHg)	0	2 (0.5)	3 (1.8)	0.032
Fasting plasma glucose (≥5.6 mmol/L)	6 (1.7)	8 (2.0)	9 (5.4)	0.027
Total cholesterol (≥5.2 mmol/L)	32 (9.0)	37 (9.2)	12 (7.2)	NS
Triglycerides (≥1.7 mmol/L)	12 (3.4)	36 (8.9)	17 (10.2)	0.002
HDL-C (<1.03 mmol/L)	87 (24.5)	149 (37.0)	71 (42.8)	<0.001
LDL-C (≥3.36 mmol/L)	89 (25.1)	118 (29.3)	52 (31.3)	NS
Presence of acanthosis nigricans	111 (31.3)	237 (58.8)	139 (83.7)	<0.001
Insulin resistance by pubertal stage	63 (19.6)	158 (43.2)	107 (72.8)	<0.001
TG:HDL-C ratio, mean (sd)	0.78 (0.45)	0.98 (0.56)	1.02 (0.50)	<0.001^a^
Metabolic syndrome	1 (0.3)	11 (2.7)	8 (4.8)	<0.001
At risk of metabolic syndrome	2 (0.6)	8 (2.0)	8 (4.8)	

Chi-squared test.

a One-way analysis of variance (ANOVA).

NS, not significant; HDL-C, high-density lipoprotein cholesterol; LDL-C, low-density lipoprotein cholesterol.

Insulin resistance is calculated using the homeostatic model assessment for IR (HOMA-IR).

The association between cardiometabolic risk factors and obesity status is shown in Table 4. Severely children affected by obesity had significantly higher odds (OR = 3.27; 95% confidence interval (CI) 1.12, 9.55) of having abnormal FPG levels compared to children affected by obesity. They also had higher odds of hypertriglyceridemia (OR = 3.50; 95%CI 1.61, 7.64), lower HDL-C (OR = 2.65; 95%CI 1.77, 3.98), the presence of acanthosis nigricans (OR = 13.49; 95%CI 8.26, 22.07), IR (OR = 14.35; 95%CI 8.84, 23.30), and MetS (OR = 14.03; 95%CI 3.97, 49.54). However, there was no significant difference in the odds of having high levels of total cholesterol and LDL-C.

**Table 4:** Association between cardiometabolic risk factors and obesity status.

**Obesity status**	**OR (95%CI)**			
	**Fasting plasma glucose (**≥**5.6 mmol/L)**	**Total cholesterol (**≥**5.2 mmol/L)**	**Triglycerides (**≥**1.7 mmol/L)**	**HDL-C (**<**1.03 mmol/L)**
Overweight	1	1	1	1
Obese	1.20 (0.41, 3.51)	1.01 (0.61, 1.67)	2.90 (1.48, 5.69)^b^	1.91 (1.38, 2.62)^c^
Severely obese	3.27 (1.12, 9.55)^a^	0.70 (0.35, 1.42)	3.50 (1.61, 7.64)^b^	2.65 (1.77, 3.98)^c^
Obesity status	OR (95%CI)			
	LDL-C (≥3.36 mmol/L)	Presence of acanthosis nigricans	Insulin resistance according to pubertal stages	At risk of metabolic syndrome and metabolic syndrome
Overweight	1	1	1	1
Obese	1.26 (0.91, 1.74)	3.53 (2.57, 4.85)^c^	3.37 (2.37, 4.81)^c^	6.03 (1.76, 20.60)^b^
Severely obese	1.46 (0.96, 2.22)	13.49 (8.26, 22.04)^c^	14.35 (8.84, 23.30)^c^	14.03 (3.97, 49.54)^c^

Multivariate logistic regression (adjusted for gender, ethnicity, and strata).

a Significant at p < 0.05;

b Significant at p < 0.01;

c Significant at p < 0.001.

OR, odds ratio; CI, confidence interval; HDL-C, high-density lipoprotein cholesterol; LDL-C, low-density lipoprotein cholesterol.

Table 5 presents the association between adiposity measures (BMI z-score, WC, and PBF) and MetS components and IR. A higher correlation was observed between adiposity measures and IR surrogate markers (HOMA-IR and TG: HDL-C ratio) compared to MetS components (*r* ≈ 0.4, *p* < 0.001 and *r* ≈ 0.2, *p* < 0.001). Additionally, a significant correlation was found between adiposity measures and triglycerides and HDL-C. All three adiposity measures were not significantly correlated with blood pressure and FPG.

**Table 5:** The correlation between adiposity (BMI z-score, WC, and PBF) and metabolic syndrome components and insulin resistance.

**Metabolic syndrome components**	**BMI** ***z*****-score**		**WC (cm)**		**PBF (%)**	
	***r***	***p*** **-value**	***R***	***p*** **-value**	***r***	***p*** **-value**
Systolic blood pressure (mmHg)	−0.0002	0.995	0.029	0.377	0.017	0.604
Diastolic blood pressure (mmHg)	0.003	0.921	0.002	0.952	0.032	0.334
Fasting plasma glucose (mmol/L)	0.068	0.040	0.0648	0.049	0.036	0.278
Triglycerides (mmol/L)	0.197	<0.001	0.252	<0.001	0.211	<0.001
HDL-C (mmol/L)	−0.177	<0.001	−0.174	<0.001	−0.155	<0.001
HOMA-IR index	0.424	<0.001	0.472	<0.001	0.414	<0.001
TG:HDL-C ratio	0.240	<0.001	0.283	<0.001	0.241	<0.001

Spearman's correlation.

BMI, body mass index; WC, waist circumference; PBF, percentage of body fat; HDL-C, high-density lipoprotein cholesterol.

According to Table 6, children affected by obesity affected by acanthosis nigricans were two times as likely to develop MetS, i.e., 2.22 (95%CI 1.09, 4.52), after adjusting for gender, ethnicity, and strata.

**Table 6:** The association between acanthosis nigricans and metabolic syndrome.

	**Metabolic syndrome and at risk of metabolic syndrome, *n* (%)**	**No metabolic syndrome, *n* (%)**	**OR (95%CI)**	***P*** **-value**
Presence of acanthosis nigricans	26 (68.4)	461 (52.0)	2.22 (1.09, 4.52)	0.028
Absence of acanthosis nigricans	12 (31.6)	425 (48.0)	1	

Adjusted for gender, ethnicity, and strata.

OR, odds ratio; CI, confidence interval.

## 4. Discussion

My Body Is Fit and Fabulous at school is a school-based lifestyle intervention program to combat obesity among overweight, obese, and severely children affected by obesity. We studied the prevalence of cardiometabolic risk factors and their association with obesity status among children using the baseline information from this intervention program. To our knowledge, this is one of the large-scale studies in Southeast Asia to embark on understanding the health-related problems among severely children affected by obesity aged 8–11 years in the community. Alarmingly, severely obese young children was almost in the proportion of 20%, which means that they were more likely to remain obese in adulthood and develop complications, such as high LDL, hypertension, and T2DM (23). A similar trend was reported in the USA. According to the National Health and Nutrition Examination Survey, the rates of obesity among children and teenage have increased to more than threefold since the 1970s, along with the rates of severe obesity increasing more than fivefold in the same period (24, 25). Therefore, it is vital to determine the clinical characteristics of this group of children. This information will give more insights into the health risk of these children. Then, the obesity strategy could be governed from prevention to early diagnosis and treatment.

It is not surprising to note that severely children affected by obesity had the highest prevalence and a greater likelihood of having cardiometabolic risk factors compared with overweight and children affected by obesity. For example, a study of 8,579 obese American children and young adults demonstrated that the mean for cardiometabolic variables and the prevalence of abnormal values were higher with greater severity of obesity, particularly triglycerides and HDL-C (26). According to a follow-up study by Morrison et al. (27), the occurrence of CVD in adulthood was highly predicted by individuals with hypertriglyceridemia in childhood. Furthermore, the association between atherosclerosis in young children and elevated BMIs was long proven by the autopsy of children and young adults from the Bogalusa Heart Study (28). Additionally, youth with traditional CVD risk factors associated with childhood hypertension (e.g., homozygous or heterozygous familial hypercholesterolemia), severe obesity, and T2DM are at a higher risk of CVD (29). Our study highlighted that a high proportion of young but obese healthy children (8–11 years) already demonstrated a poor cardiometabolic profile, which could potentially lead to premature cardiovascular events if left unattended (18).

We also found a higher prevalence and greater likelihood of having acanthosis nigricans among severely children affected by obesity. The increase in adipose tissues during childhood is a plausible starter to develop abnormal glucose metabolism associated with IR (30). This supports the presence of acanthosis nigricans as one of the clinical markers of IR in children affected by obesity (31). Our study showed that children affected by obesity with acanthosis nigricans were two times as likely to have MetS, which was consistent with a few other reported studies among children (32, 33). This observation is important as a cohort study by Morrison et al. (34) found that 10-year-old girls diagnosed with both MetS and hyperinsulinemia had a high frequency of progression to T2DM 14 years later.

Along with obesity, the prevalence of MetS has increased worldwide. Its prevalence affects not only adults but also children (4). In this study, we chose the IDF 2007 definition as the definition of MetS for children that are currently being used worldwide, stratified by age with fixed cutoffs for blood pressure, lipids, glycemia, and abdominal circumferential points assessed by percentile (35). Additionally, the presence of MetS using this definition is predictive of an increased cardiometabolic risk (36). Surprisingly, 4.8% of severely children affected by obesity younger than 10 years of age were already at risk of having MetS. The percentage was comparable to that of severely children affected by obesity who were 10 years of age and over and had MetS. Fortunately, this prevalence is much lower than that reported in a school-based study by Palhares et al. (37), with 13.6% of obese Brazilian children having MetS (using the same definition of MetS).

In addition, our findings showed that all adiposity markers, i.e., BMI, WC, and PBF, were significantly correlated with the HOMA-IR, TG: HDL-C ratio, triglycerides, and HDL-C. Interestingly, although there was no correlation with FPG, the highest correlation was observed with HOMA-IR, suggesting that childhood obesity studies should venture into IR beyond glucose measurements. Previous studies also showed that most obese and IR individuals did not develop hyperglycemia (38). However, the association between IR and childhood obesity is beyond the scope of this article. Moreover, a non-significant correlation with MetS components, such as high LDL-C and hypertension, could be explained by the age of the children involved in this study. This study involved young children in the early years of obesity. Therefore, complications such as high LDL-C, hypertension, and diabetes were not yet observed as these diseases were time-sensitive (depending on the chronic level of obesity).

The major strength of this study is that it is a community-based study with a large data set, with a huge number of students being screened, and with ages as early as 8 years old. In addition, our study included severely children affected by obesity, while other studies tended to exclude them. As proven in the present study, severely children affected by obesity were more likely to have cardiometabolic risk factors. Additionally, the HOMA-IR cutoff was adopted based on pubertal stages, considering the influence of the pubertal state with IR (39, 40). Another strength of this study was that proportionate random sampling was used to select schools to ensure sufficient representation of multiethnic and socioeconomically diverse populations in Malaysia. An adjustment for potential confounding factors was also done for the association between cardiometabolic risk factors and obesity status.

Nevertheless, this study has some limitations. One limitation is that we do not have adequate information on the socioeconomic status despite attempting our best to obtain the parental monthly income. However, we do have the information on strata, either from the urban or rural school, which broadly indicates their socioeconomic background. Hypertension could not be further analyzed as there were very few children with hypertension. It is impossible to explain the causal relationship between obesity markers and a cardiometabolic risk as this study only used the baseline data from an intervention study. In essence, we recommend initiating a cohort study to investigate the association further.

## 5. Conclusion

This study has demonstrated that severely obese young children aged 8–11 years have a significantly greater cardiometabolic risk, which may lead to premature cardiovascular complications, such as myocardial infarction, or cerebrovascular accidents. This finding is particularly important in managing childhood obesity, and it is therefore essential to implement effective intervention programs for severely children affected by obesity. These young children require early and targeted intervention. Additionally, obesity-related health problems should be monitored closely and screened periodically to evaluate both prevention and treatment strategies.

## Data availability statement

The raw data supporting the conclusions of this article will be made available by the authors, without undue reservation.

## Ethics statement

The studies involving human participants were reviewed and approved by Medical Research and Ethics Committee (MREC), Ministry of Health Malaysia. Written informed consent to participate in this study was provided by the participants' legal guardian/next of kin. The assent form was signed by the participating child.

## Author contributions

AHM as a principal investigator and coordinator of this study. AHM, MYJ, FMZ, RS, and ZI contributed to the overall concept and design of the study. MYJ, FMZ, JYHH, and FM contributed to the concept of the clinical part of the study. FM, RMWMZ, AKNZI, and FAR contributed to the logistics and sample collection. FAR contributed to the laboratory analysis. FAR, AY, RMWMZ, and AKNZI contributed to the data management and analysis. All authors read and approved the manuscript.
